# Editorial Notes

**Published:** 1934

**Authors:** 


### Editorial Notes

**Index to Volumes 1–50.**

At last the Index to our first fifty volumes is complete. It is the product of the joint labours of the Assistant Editor (Mr. E. Watson-Williams) and the Senior Assistant in the University Library (Mr. A. E. S. Roberts). The value of such an index is great, although the number of users may be small. From time to time some research worker has occasion to refer to the work of his forerunners, and a comprehensive index is then indispensable.

A journal such as ours contains references to current events which as time passes become of increasing importance to historians. When contemporary records have to be searched through the boon of a good index cannot be over-estimated. This fifty-volume index is long and has proved a costly undertaking, but our subscribers will not grudge the cost, even as the compilers have not grudged their labour. "Time is of more value than type and the wear and tear of temper than an extra page of index."

**Winford Orthopædic Hospital.**

The Winford Orthopædic Hospital has sustained a great loss owing to the resignation of its Chairman, Miss F. M. Townsend, M.A., J.P. For many years Miss Townsend has been prominent in this city in all educational work, and in particular has been closely associated with the welfare and education of the mentally and physically defective.

She founded the Knowle Open Air School, the first of its kind in the city, and at first a voluntary organization, though later taken over by the Education Committee. She has also been chairman of the committee dealing with Special Schools.

Miss Townsend was the prime mover in the foundation of the Crippled Children's Aid Society, and when it was decided to move the Orthopædic Hospital from Grove Road into the country it was chiefly owing to her energy and organizing ability that the Winford Orthopædic Hospital came into existence. That this was recognized by all was shown by the unanimity with which she was elected its first chairman.

It must be a matter of profound regret to everyone that ill-health should have compelled her to vacate a post which she had so ably filled.

Her place is being taken by Sir Lionel Goodenough Taylor, who has long been intimately associated with the Grove Road Hospital and with its successor at Winford. Of the latter he has been Treasurer and has also acted as Chairman since Miss Townsend fell ill, so that no one could be better fitted to take on the work which Miss Townsend has been so unfortunately compelled to abandon.

